# High energy storage density over a broad temperature range in sodium bismuth titanate-based lead-free ceramics

**DOI:** 10.1038/s41598-017-06966-7

**Published:** 2017-08-18

**Authors:** Haibo Yang, Fei Yan, Ying Lin, Tong Wang, Fen Wang

**Affiliations:** School of Materials Science and Engineering, Shaanxi University of Science and Technology, Weiyang, Xi’an, Shaanxi 710021 PR China

## Abstract

A series of (1-x)Bi_0.48_La_0.02_Na_0.48_Li_0.02_Ti_0.98_Zr_0.02_O_3_-xNa_0.73_Bi_0.09_NbO_3_ ((1-x)LLBNTZ-xNBN) (x = 0-0.14) ceramics were designed and fabricated using the conventional solid-state sintering method. The phase structure, microstructure, dielectric, ferroelectric and energy storage properties of the ceramics were systematically investigated. The results indicate that the addition of Na_0.73_Bi_0.09_NbO_3_ (NBN) could decrease the remnant polarization (*P*_*r*_) and improve the temperature stability of dielectric constant obviously. The working temperature range satisfying *TCC*_150 __°C_ ≤±15% of this work spans over 400 °C with the compositions of x ≥ 0.06. The maximum energy storage density can be obtained for the sample with x = 0.10 at room temperature, with an energy storage density of 2.04 J/cm^3^ at 178 kV/cm. In addition, the (1-x)LLBNTZ-xNBN ceramics exhibit excellent energy storage properties over a wide temperature range from room temperature to 90 °C. The values of energy storage density and energy storage efficiency is 0.91 J/cm^3^ and 79.51%, respectively, for the 0.90LLBNTZ-0.10NBN ceramic at the condition of 100 kV/cm and 90 °C. It can be concluded that the (1-x)LLBNTZ-xNBN ceramics are promising lead-free candidate materials for energy storage devices over a broad temperature range.

## Introduction

In order to meet the increasing demand for electrical energy storage capacitors in the field of pulse power applications, especially dielectric ceramic capacitors for high energy storage density devices, have been widely investigated and played more and more important roles^1–4^. Compared with batteries and other energy storage devices, ceramic capacitors possess faster charge-discharge rate, superior mechanical and thermal properties^5–7^. However, the energy that ceramic capacitors can store is much less than those of the fuel cells or lithium ion batteries^8, 9^. Recently, for the needs of practical application, growing interests have been attracted on dielectrics, which have stable energy storage properties over a brode temperature range. Thus, the development of ceramic capacitor materials with high energy storage density and broad working temperature range is a challenge for researchers^10^.

Due to the excellent properties, PbZr_x_Ti_(1−x)_O_3_ (PZT) and other lead-containing materials such as Pb(Zn_1/3_Nb_2/3_)O_3_-Pb_0.96_La_0.04_(Zr_x_Ti_1−x_)_0.99_O_3_ are often the materials of choice^11, 12^. However, for the sake of environment protection, these lead-containing materials need to be replaced by environment-friendly materials. As an alternative to the toxic lead-containing dielectric materials, Bi_0.5_Na_0.5_TiO_3_ (BNT) based ceramics have been extensively studied^13–18^, such as (Bi_0.5_Na_0.5_)TiO_3_-BaTiO_3_^19–21^, (Bi_0.5_Na_0.5_)TiO_3_-BaTiO_3_-(K_0.5_Na_0.5_)NbO_3_^22^, (Na_0.5_Bi_0.5_)TiO_3_-SrTiO_3_^23^ and Na_0.5_Bi_0.5_TiO_3_-BaTiO_3_-BiFeO_3_^24^. The reason is that BNT belongs to perovskite-type ferroelectric with an A-sites disorder structure and Bi^3+^ ion is a promising alternative to Pb^2+^ ion due to their similar lone-pair electronic *6* *s* ^*2*^ configuration^1, 25^. Generally, the dielectric property of BNT shows three dielectric anomalies with increasing the temperature^26^. They are the shoulder with a strong frequency dependent of dielectric constant anomaly at ~200 °C, the peak with a broad dielectric constant maximum at ~325 °C, and the hump of dielectric loss (depolarization temperature, *T*_*d*_) at a low temperature of ~190 °C^26–29^. The double dielectric constant peaks for BNT-based ceramics can be modified by the introduction of other components^26–28, 30^. It is beneficial to improve the temperature stability of dielectric constant over a broad temperature range. Meanwhile, it can be seen that the temperature stability of dielectric constant is beneficial to improve the temperature stability of energy storage density according to Equation (1).

1 $${W}_{1}={\int }_{0}^{E}{\varepsilon }_{0}\varepsilon ^{\prime} EdE$$

where *W*_*1*_ is the energy storage density, *ε*_0_ is the dielectric constant of free space (8.854 × 10^−12^ F/m), *ε*′ is the dielectric constant of materials and E is applied electric field (kV/cm). In addition, the occupation of Li^+^ and La^3+^ ions in A-sites tend to shrink the lattice owing to the formation of oxygen vacancies and smaller ionic sizes of Li^+^ and La^3+^ than those of Na^+^ and Bi^3+^ (ionic radii: 1.39, 1.38, 1.36, and 0.92 Å for Na^+^, Bi^3+^, La^3+^, and Li^+^)^26, 31, 32^. It is well known that Ti^4+^ and Zr^4+^ possess the identical valence, but Zr^4+^ has more chemical stablity and larger ionic size. So the substitution of Zr for Ti would decrease the leakage current, induce good thermal stability and depress the conduction caused by hopping between Ti^4+^ and Ti^3+^ ^33, 34^. Moreover, BNT-based ceramics have been recognized as potential lead-free ferroelectric materials owing to their large saturated polarization. Therefore, BNT-based ceramics have a great potential to enhance energy storage properties while the energy storage density is limited due to large remanent polarizations (*P*_*r*_)^35, 36^. Thus, it is very important to reduce the value of *P*_*r*_ and improve the thermal stability of BNT-based ceramics for their applications in energy storage capacitors. Generally speaking, the energy storage density and the temperature stablity of BNT-based ceramics can be improved by Nb doping or the addition of niobates^16, 37–39^. However, there are few reports of temperature stability on the energy storage and dielectric properties for BNT-based ceramics that modified by Na_0.73_Bi_0.09_NbO_3_.

In the present study, in order to obtain a high energy storage density and an excellent dielectric temperature stability, a new lead-free ferroelectric solid solution of (1-x)LLBNTZ-xNBN ceramics were reported. Its phase structure, microstructure, dielectric, ferroelectric and energy storage properties were systematically investigated. The results demonstrate that the (1-x)LLBNTZ-xNBN ceramics are promising for energy storage application over a broad temperature range.

## Results and Discussion

**Figure 1:**
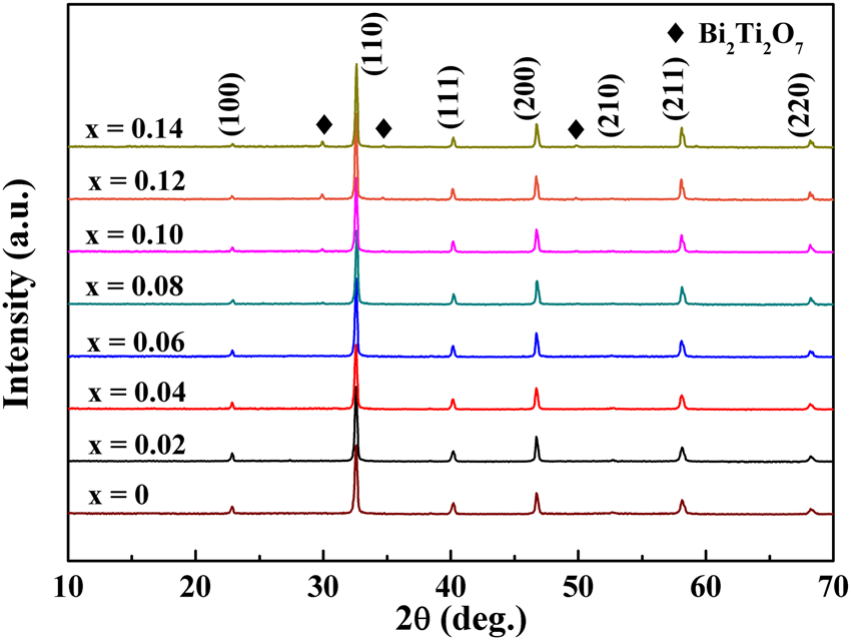
XRD patterns of the (1-x)LLBNTZ-xNBN ceramics.

The X-ray diffraction (XRD) patterns of the (1-x)LLBNTZ-xNBN ceramics with the value of x from 0 to 0.14 recorded at room temperature are illustrated in Fig. 1. It can be observed that single perovskite phase are formed for the compositions of x < 0.10, while a small amount of secondary phase appears for the compositions of x ≥ 0.10 according to the analysis result of XRD data by MDI Jade software. The results show that the minor secondary phase is Bi_2_Ti_2_O_7_, which is a linear dielectric and also found in other BNT-based systems^40–43^. The formation of the secondary phase may be related to the deficiency of Na and Bi in the system^16, 40^. The lattice parameters calculated from the XRD patterns are plotted in Fig. S1 and the inset figure shows the composition dependence of the unit cell volume. It can be seen that the lattice parameters and unit cell volume exhibit tiny fluctuations in the range of x from 0 to 0.14, which indicates that the (1-x)LLBNTZ-xNBN ceramics have a stable monoclinic structure for all the samples at room temperature.

**Figure 2:**
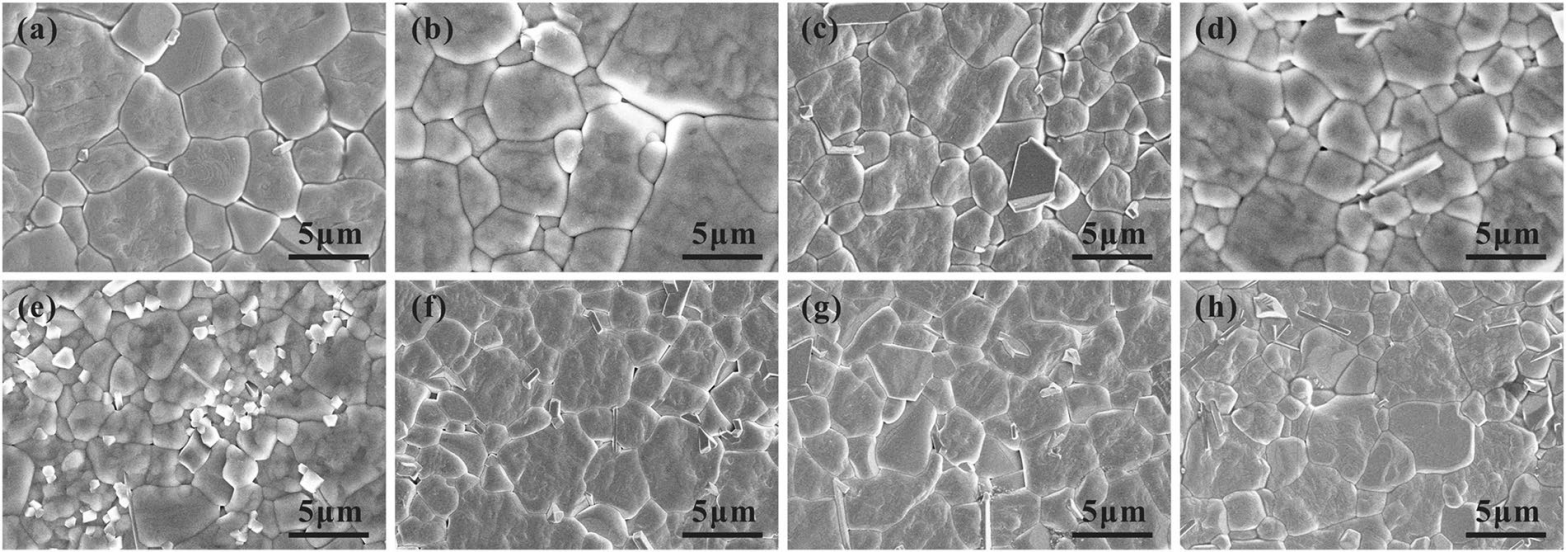
SEM micrographs on the polished and thermal-etched surfaces of the (1-x)LLBNTZ-xNBN ceramics: (**a**) x = 0, (**b**) x = 0.02, (**c**) x = 0.04, (**d**) x = 0.06, (**e**) x = 0.08, (**f**) x = 0.10, (**g**) x = 0.12, (**h**) x = 0.14.

Figure 2 shows the typical surface scanning electron microscopy (SEM) morphology of the polished and thermally etched samples for the (1-x)LLBNTZ-xNBN ceramics with different values of x. It can be seen that all the (1-x)LLBNTZ-xNBN ceramics are densely sintered with a homogeneous grain size and few visible pores appear. In order to easily identify the average grain size of the samples, the average grain size of (1-x)LLBNTZ-xNBN ceramics was calculated by a linear interception method using an analytical software (Nano Measurer) and the results are shown in Fig. S2. It can be seen that the average grain size of (1-x)LLBNTZ-xNBN ceramics decreases with increasing the x value firstly and then almost keeps unchanged with further increasing the x value when x ≥ 0.10. A small quantity of secondary phase can be found when x ≥ 0.06, which is similar with the above XRD analysis results. However, the slight difference between the XRD results and SEM results is due to the fact that the secondary phase is too small to be detected by XRD for the samples with the x values of 0.06 and 0.08.

**Figure 3:**
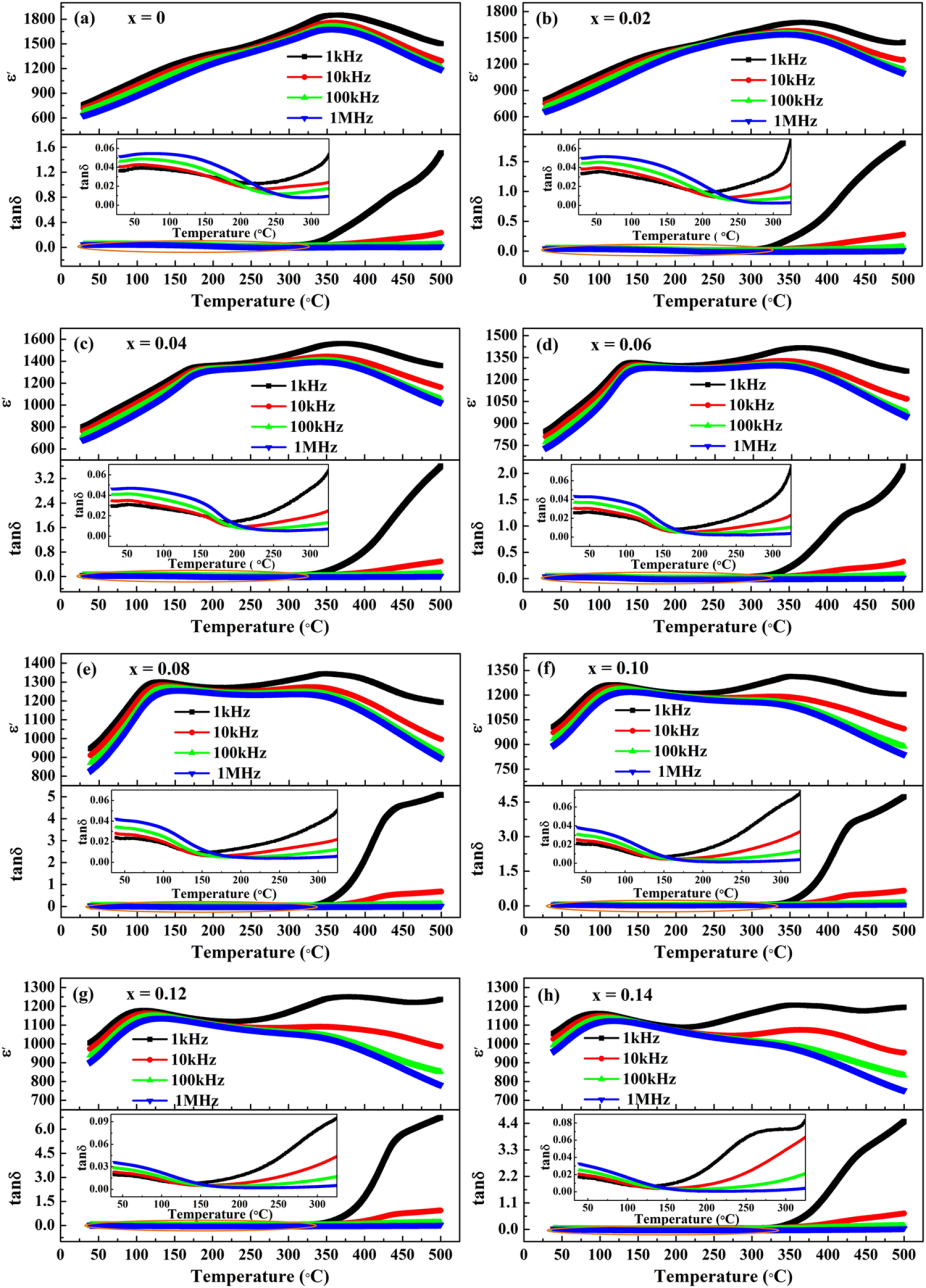
Temperature dependence of dielectric constant and dielectric loss of the (1-x)LLBNTZ-xNBN ceramics measured from 1 kHz to 1 MHz: (**a**) x = 0, (**b**) x = 0.02, (**c**) x = 0.04, (**d**) x = 0.06, (**e**) x = 0.08, (**f**) x = 0.10, (**g**) x = 0.12, (**h**) x = 0.14. (The inset shows dielectric loss of the (1-x)LLBNTZ-xNBN ceramics from room temperature to 325 °C.)

**Figure 4:**
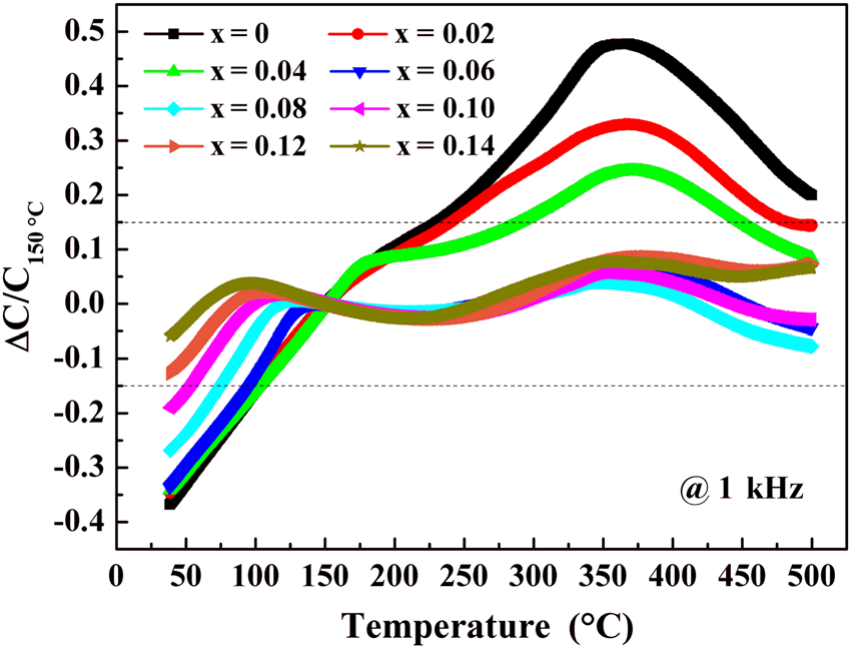
*TCC* (base temperature 150 °C) of the (1-x)LLBNTZ-xNBN ceramics at 1 kHz.

The temperature dependence of dielectric constant and dielectric loss of unpoled (1-x)LLBNTZ-xNBN ceramics from room temperature to 500 °C with different frequencies are displayed in Fig. 3. It can be seen that the temperature dependent of dielectric constant curves are characterized by double dielectric constant anomalies, attributed to the presence of two types of polar nano-regions (PNRs) (low-temperature PNRs (LT-PNRs) and high-temperature PNRs (HT-PNRs))^44–48^. The origin of dielectric anomalies can be regarded as a convolution of three distinctive processes as follows^47, 49^: (a) Low temperature: a dielectric relaxation contributed by coexisting PNRs of different symmetries, (b) intermediate temperature: a diffuse phase transition from low symmetry PNRs to higher symmetry ones and (c) high temperature: an additional dielectric relaxation process from the remaining high symmetry PNRs. The first dielectric anomaly is located at a low temperature of ~170 °C (Fig. 3 (c)) and shows an obvious frequency dispersion, which is caused by the thermal evolution of *R3c* and *P4bm* PNRs coexisted in a wide temperature range for BNT-based ceramics^10, 47, 50^. The second dielectric constant anomaly is located at a higher temperature of ~360 °C (Fig. 3(c)), which is also regarded as the Curie temperature (*T*_*C*_) and known to arise from a convolution of symmetry change in PNRs and a relaxation of HT-PNRs^51, 52^. For the temperature dependent dielectric loss curves, a peak in temperature range of 30 °C–150 °C can be observed for all the samples, which is often defined as the depolarization temperature (*T*_*d*_) of the system^30^. Meanwhile, the dielectric loss is lower than 0.09 in the temperature range from room temperature to 325 °C. Temperature dependent dielectric constant and dielectric loss measured at 1 kHz for different compositions are summarized in Fig. S3. It can be found that the first dielectric constant anomaly peak shifts to lower temperature and the magnitude of the anomaly peak decreases with increasing the value of x. It is due to the fact that large differences of ion valences and sizes among Ti^4+^, Zr^4+^ and Nb^5+^ in B-sites disturb the long range ferroelectric order of ceramics^10, 53, 54^. The second dielectric constant anomaly peak also decreases in magnitude while its position remains almost unchanged. This phenomenon is beneficial to improve the temperature stability of dielectric constant. We adopted temperature coefficient of capacitance (*TCC*) to evaluate the temperature stability of dielectric properties for (1-x)LLBNTZ-xNBN ceramics, as shown in Equation (2)^10^.

2 $$TCC=\frac{{\rm{\Delta }}C}{{C}_{BaseTemp.}}=\frac{{C}_{T}-{C}_{BaseTemp.}}{{C}_{BaseTemp.}}$$

where *C*_*T*_ represents the capacitance at certain temperature within the measuring range, *C*_*Base Temp*._ is the capacitance at the base temperature. The variance of *TCC* for (1-x)LLBNTZ-xNBN ceramics at 1 kHz as a function of temperature is presented in Fig. 4 and the dashed lines indicate the operational ranges within ±15%. The value of capacitance at 150 °C is regarded as the reference point, since it is the midpoint of the desired operational temperature range and has also been used for other NBT-based high temperature dielectrics^54, 55^. As shown in Fig. 4, the sample of x = 0 has a narrow working temperature range of *TCC* and the working temperature range of *TCC* is expanded with increasing the value of x gradually for (1-x)LLBNTZ-xNBN ceramics. The working temperature range satisfying *TCC*_150 °C_ ≤ ± 15% of this work spans over 400 °C with the compositions of x ≥ 0.06. A comparison of the temperature stability of dielectric constant between (1-x)LLBNTZ-xNBN ceramics and other reported lead-free ceramics in literatures are list in Table S1. It can be seen that the temperature stability of dielectric constant for (1-x)LLBNTZ-xNBN ceramics is superior to the previously reported results.

**Figure 5:**
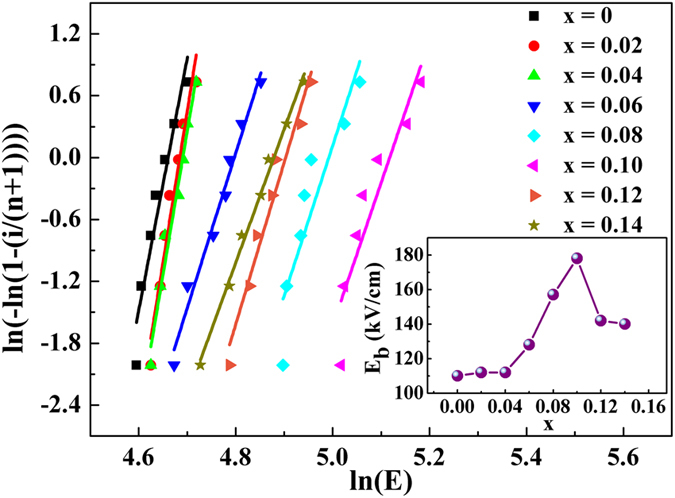
Weibull distribution of BDS for the (1-x)LLBNTZ-xNBN ceramics. (The inset shows BDS as a function of x for the (1-x)LLBNTZ-xNBN ceramics).

Figure 5 shows the Weibull distribution of the (1-x)LLBNTZ-xNBN ceramics, which is usually used for BDS analysis due to its statistical nature of failure^56–58^. The plot is described as shown in the following equations^59–61^.

3 $${X}_{i}=\,\mathrm{ln}({E}_{i})$$

4 $${Y}_{i}=\,\mathrm{ln}(-\mathrm{ln}(1-\frac{i}{n+1}))$$

where *X*_*i*_ and *Y*_*i*_ are two important parameters for Weibull distribution function. *E*_*i*_ is specific breakdown voltage of each sample in the experiments. *i* is serial number of samples and *n* is the sum of samples. The samples are arranged in ascending order of BDS values so that:

5 $${E}_{1}\le {E}_{2}\le {E}_{3}\,\le \,\cdots \,\le {E}_{i}\,\le \,\cdots \,\le {E}_{n}$$

The values of BDS for the (1-x)LLBNTZ-xNBN ceramics are obtained and shown in the inset of Fig. 5. It can be seen that the BDS values increase first and then decrease with increasing the value of x. And the maximum BDS value of 178 kV/cm occurs when x = 0.10. It is well known that dielectric ceramics with small and homogeneous grain size usually exhibit high BDS^62, 63^. Thus, it is believed that the improvement of the BDS is attributed to the decreased average grain size for the (1-x)LLBNTZ-xNBN ceramics.

**Figure 6:**
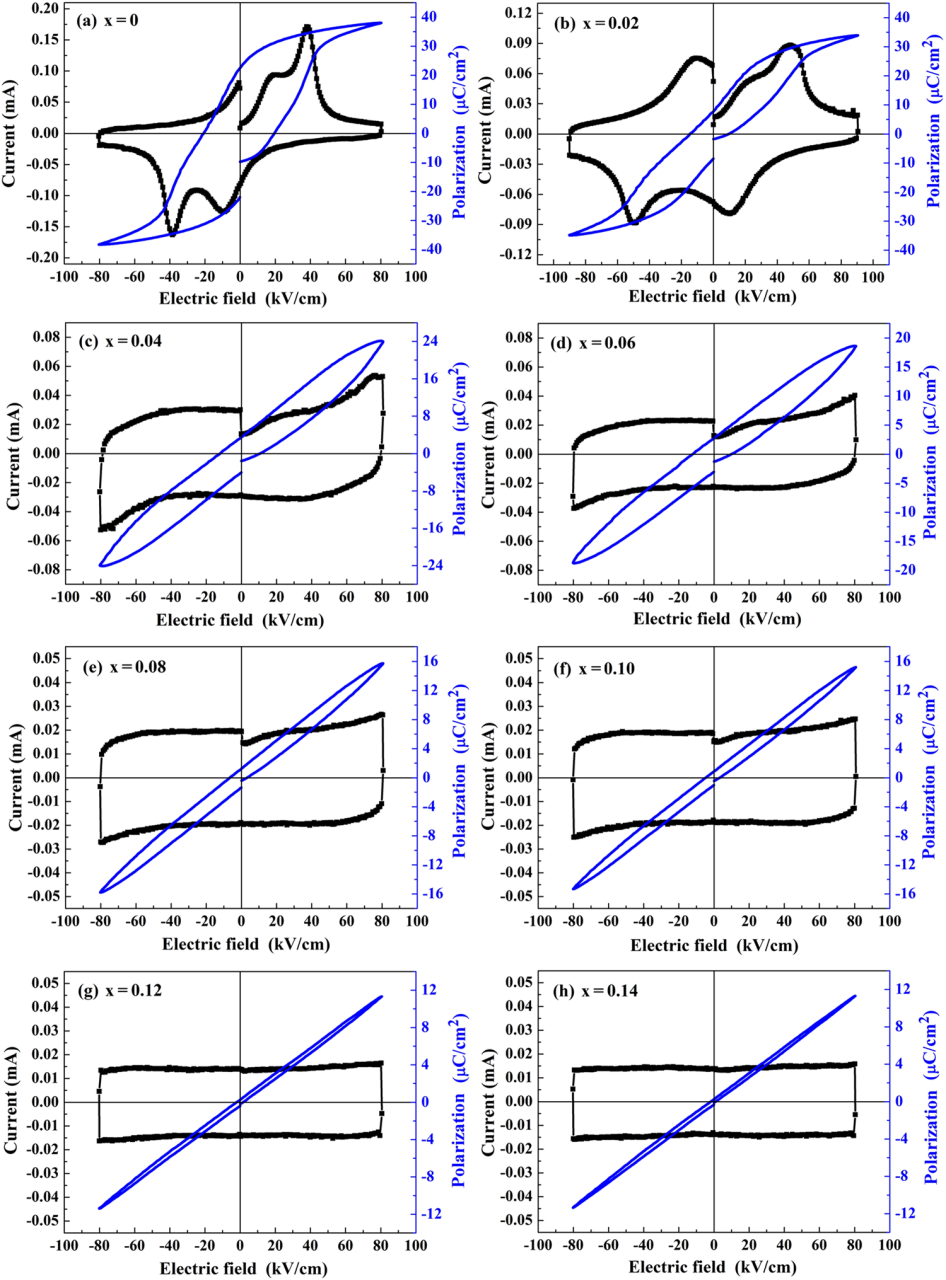
*P-I-E* plots for the (1-x)LLBNTZ-xNBN ceramics at 90 °C and 10 Hz.

To investigate the influence of NBN addition on the ferroelectric and energy storage properties of the (1-x)LLBNTZ-xNBN ceramics, *P-I-E* characteristics were measured at the condition of 80 kV/cm, 10 Hz and 90 °C, as shown in Fig. 6(a)–(h). It is found that the *P-E* loops display a saturated ferroelectric behavior with large values of *P*_*r*_ and maximum polarization (*P*_*max*_) when x = 0. Four current peaks emerge in the *I-E* loops when x = 0 and x = 0.02. Meanwhile, there are two current peaks with either positive or negative field for the (1-x)LLBNTZ-xNBN ceramics when x = 0 and x = 0.02, which indicates that the (1-x)LLBNTZ-xNBN ceramics have double hysteresis loops^64^. With increasing the value of x, slimmer and slimmer *P-E* loops are achieved with decreased *P*_*r*_, accompanied by slant rectangle-like *I-E* loops without obvious current peaks. It can be supposed that the ferroelectric order is disturbed, leading to a transformation from classical ferroelectric state to relaxor state for the (1-x)LLBNTZ-xNBN ceramics with increasing the value of x. The similar phenomenon was also reported in other BNT-based ceramics^14, 65^.

**Figure 7:**
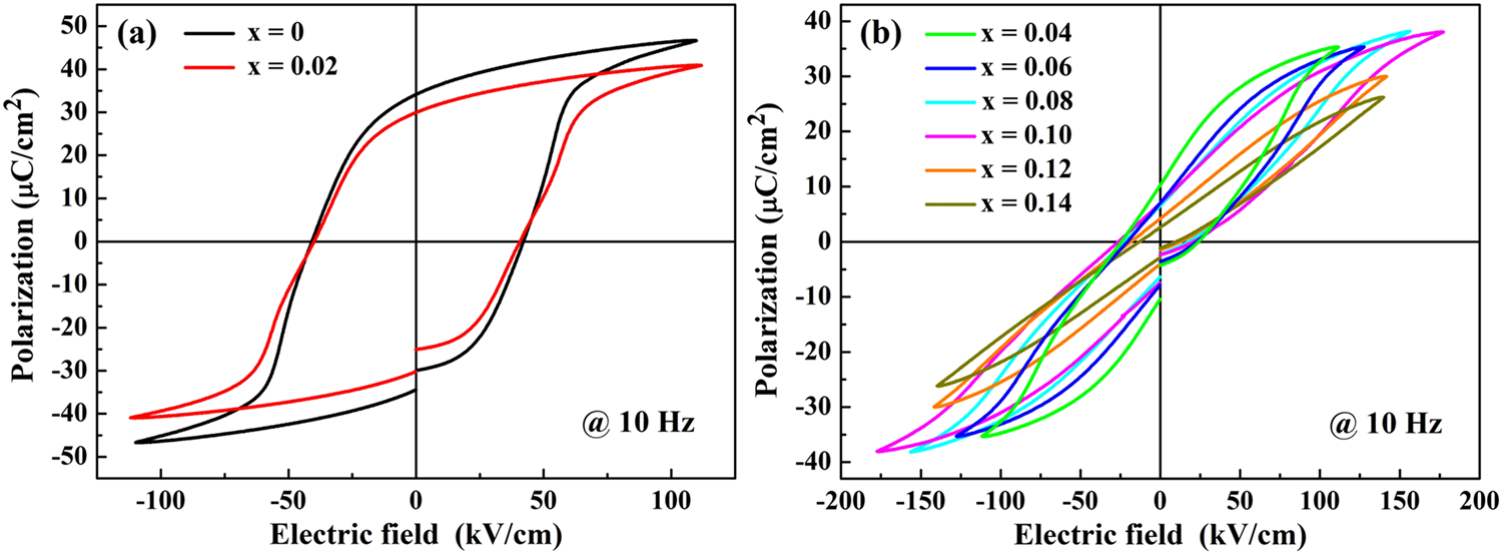
*P-E* loops of the (1-x)LLBNTZ-xNBN ceramics measured at room temperature: (**a**) x = 0–0.02, (**b**) x = 0.04–0.14.

Figure 7 shows the *P-E* loops for the (1-x)LLBNTZ-xNBN ceramics at room temperature and 10 Hz just under the electrical field of dielectric breakdown strength. It can be seen that *P*_*max*_ decreases with increasing the value of x. The (1-x)LLBNTZ-xNBN ceramics possess well-saturated *P-E* loops and large remanent polarizations (*P*_*r*_) when x = 0. It is due to the fact that the easily induced irreversible transformation from non-ergodic nano-domain PNRs to the normal ferroelectric phase^66^. As is evident in Fig. 7, NBN exerts a significant influence on the shape and polarization values of the *P-E* loops, especially when x ≥ 0.04. It can be seen that the value of *P*_*r*_ decrease gradually with increasing the x value. This can be attributed to the fact that the destabilization of long-range order occurs^66–68^. The variation of *P*_*r*_ and *P*_*max*_ as a function of the composition is shown in Fig. S4. It can be observed that *P*_*r*_ and *P*_*max*_ both decreases with increasing the value of x.

**Figure 8:**
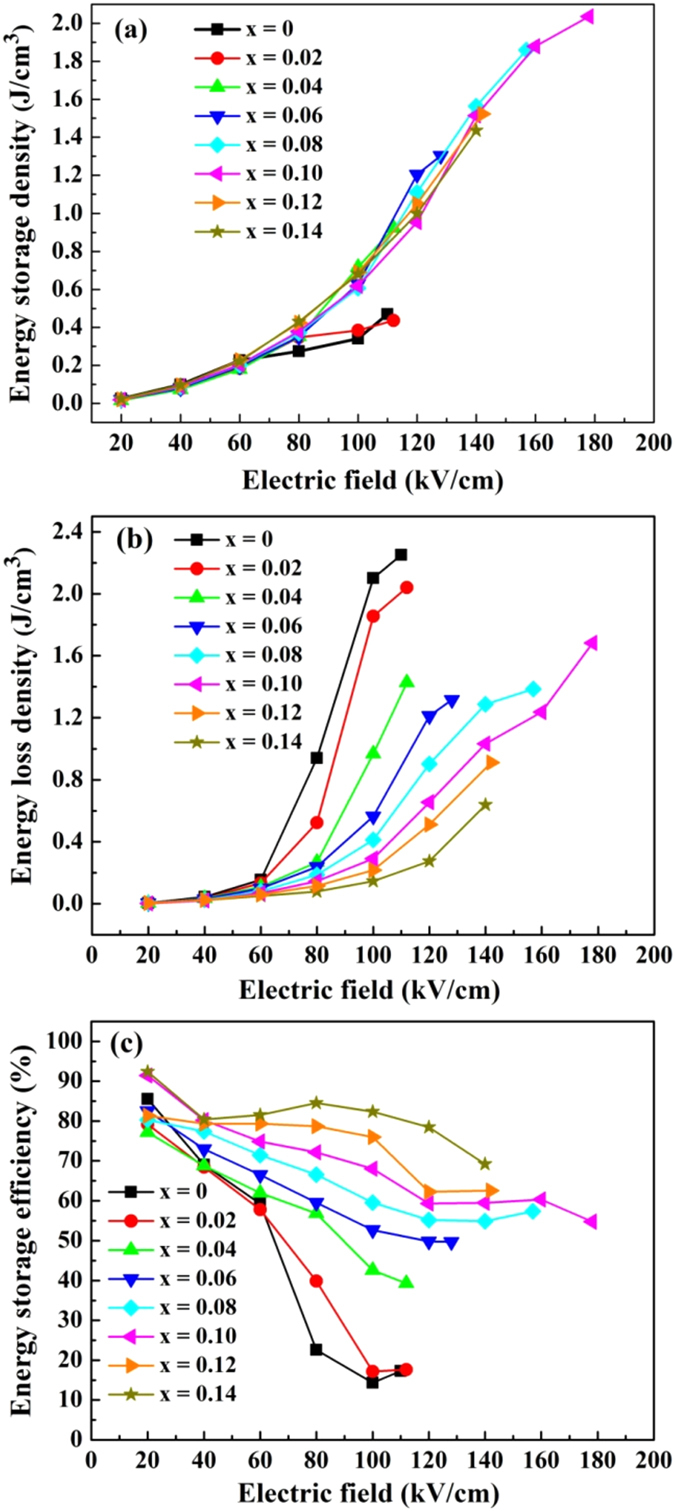
Calculated energy storage density, energy loss density and energy storage efficiency as a function of electric field for the (1-x)LLBNTZ-xNBN ceramics at room temperature.

**Figure 9:**
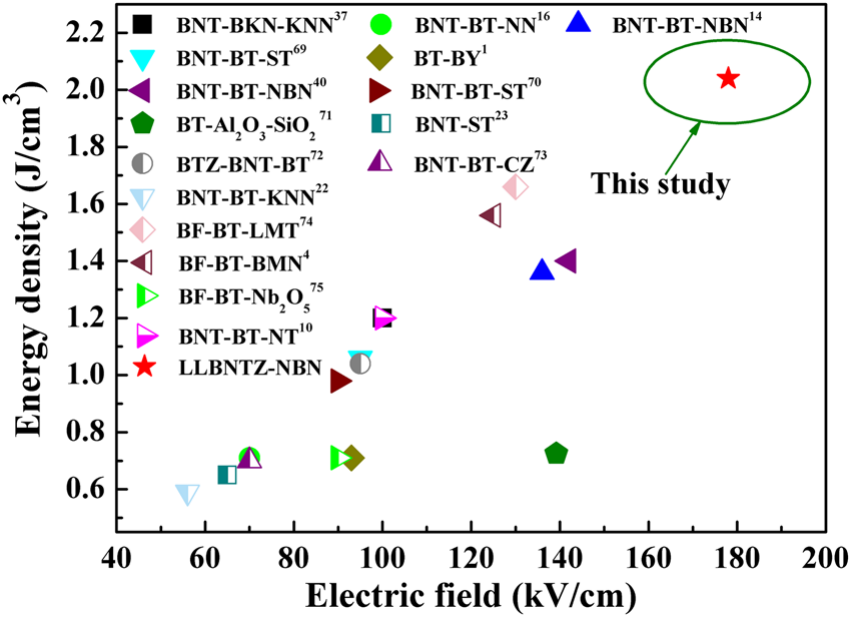
Comparison of *W*_*1*_ and BDS between (1-x)LLBNTZ-xNBN ceramics and other lead-free ceramics. (BNT: Bi_0.5_Na_0.5_TiO_3_, BKT: Bi_0.5_K_0.5_TiO_3_, KNN: K_0.5_Na_0.5_NbO_3_, BT: BaTiO_3_, NN: NaNbO_3_, NBN: Na_0.73_Bi_0.09_NbO_3_, ST: SrTiO_3_, BY: BiYbO_3_, BTZ: BiTi_0.5_Zn_0.5_O_3_, CZ: CaZrO_3_, BF: BiFeO_3_, LMT: La(Mg_0.5_Ti_0.5_)O_3_, BMN: Ba(Mg_1/3_Nb_2/3_)O_3_, NT: NaTaO_3_, LLBNTZ: Bi_0.48_La_0.02_Na_0.48_Li_0.02_Ti_0.98_Zr_0.02_O_3_).

Because of their hysteretic *P-E* loops, wherein the charging and discharging paths are not coincident, energy delivered to the capacitor cannot be released completely. Therefore, energy storage density, energy loss density and energy storage efficiency are important metrics to benchmark dielectrics for use in energy storage devices. For practical applications, energy storage density, energy loss density and energy storage efficiency should be taken into consideration^1^. Usually, the energy storage density (*W*_*1*_), energy loss density (*W*_*2*_) and the energy storage efficiency (*η*) are calculated using Equations (6), (7) and (8), respectively.

6 $${W}_{1}={\int }_{{P}_{r}}^{{P}_{\max }}EdP$$

7 $${W}_{2}={\int }_{0}^{{P}_{\max }}EdP-{W}_{1}$$

8 $$\eta =\frac{{W}_{1}}{{W}_{1}+{W}_{2}}\times 100 \% $$

where *E* is the applied electric field and *P* is polarization, whereas *P*_*max*_ is the maximum polarization with respect to the maximum experimental electric field. *W*_*2*_ is the energy loss density caused by the domain reorientation. In general, the energy densities can be obtained from *P-E* hysteresis loops. *W*_*1*_ can be evaluated by integrating the area between the polarization axis and the discharge curve, and *W*_*2*_ is obtained by integrating the area between the charge and discharge curve. Schematic diagram for the calculation of energy storage properties based on the *P-E* loop of the (1-x)LLBNTZ-xNBN ceramics is shown in Fig. S5. The energy storage efficiency is the ratio of discharge energy density to charge energy density and the area of the loop represents the energy loss density. Calculated energy storage density, energy loss density and energy storage efficiency as a function of electric field for the (1-x)LLBNTZ-xNBN ceramics at room temperature are shown in Fig. 8. The numerical values are listed in Table S2. It can be seen that the energy storage density and the energy loss density increase with increasing the electric field (Fig. 8(a) and (b)), while the energy storage efficiency decrease with increasing the electric field (Fig. 8(c)), which originates from a dramatic increase in the energy loss density. Meanwhile, the energy loss density of the (1-x)LLBNTZ-xNBN ceramics decrease while energy storage density increase first and then drop with increasing the value of x. The 0.90LLBNTZ-0.10NBN ceramic exhibits the highest energy storage density, which reaches up to 2.04 J/cm^3^ at 178 kV/cm. It is due to the fact that the BDS values increase first and then decrease with increasing the value of x for (1-x)LLBNTZ-xNBN ceramics. In addition, the (1-x)LLBNTZ-xNBN ceramics maintain a large value of *P*_*max*_ (38 μC/cm^2^) and possesses a small value of *P*_*r*_ (6.96 μC/cm^2^) when x = 0.10. For further evaluating energy storage performance of the (1-x)LLBNTZ-xNBN ceramics, the comparison of *W*_*1*_ and BDS between (1-x)BBNT-xNBN ceramics and other lead-free ceramics in recently reported results are shown in Fig. 9 ^1, 4, 10, 14, 16, 22, 23, 37, 40, 69–75^. It can be seen that the values of *W*_*1*_ and BDS for (1-x)LLBNTZ-xNBN ceramics are higher than those of other lead-free ceramics.

**Figure 10:**
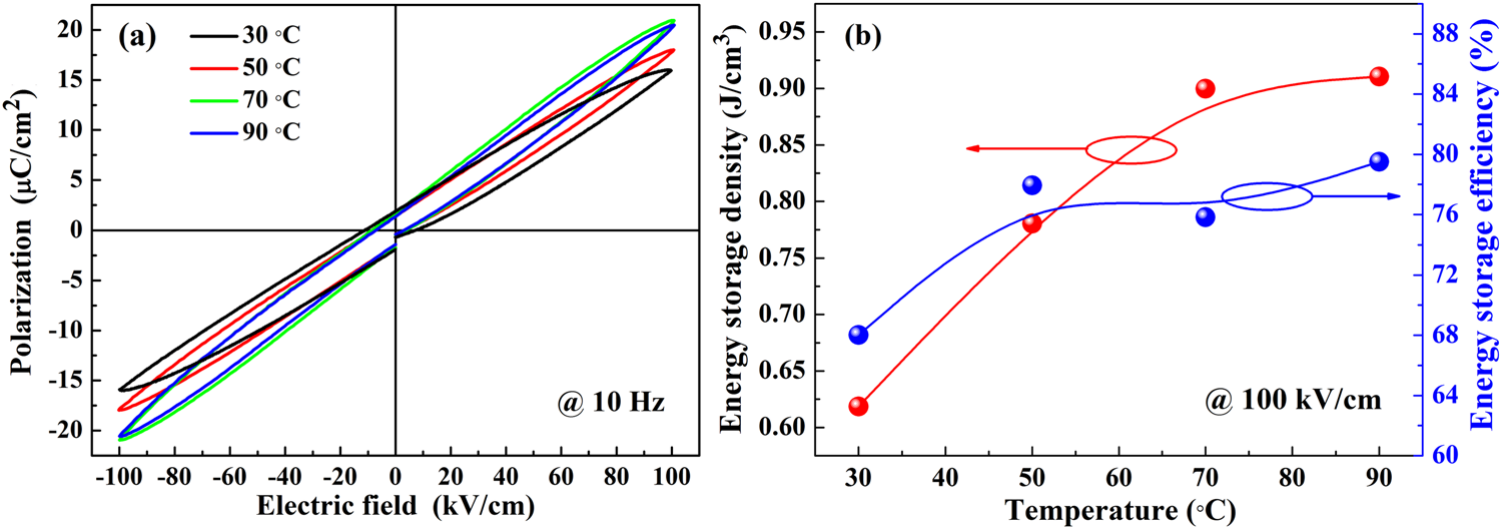
(**a**) *P-E* loops of the 0.90LLBNTZ-0.10NBN ceramic under different temperatures. (**b**) Temperature dependent energy storage density and energy storage efficiency of the 0.90LLBNTZ-0.10NBN ceramic.

Figure 10(a) reveals *P-E* loops of the 0.90LLBNTZ-0.10NBN ceramic at 100 kV/cm under different temperatures. A slow and slight increase in the *P*_*max*_ value can be seen with increasing the temperature, indicating that ergodic and/or nonergodic relaxor states could be transformed into a long-range ferroelectric order^4^. A gradual decrease in *P*_*r*_ and the *P-E* loops become slimmer and slimmer with increasing the temperature, which is attributed to the decrease of content of nonergodic phase^4^. Therefore, the gradual decrease in *P*_*r*_ and increase in *P*_*max*_ are beneficial to improve energy storage properties over a broad temperature range. The energy storage density and energy storage efficiency for the 0.90LLBNTZ-0.10NBN ceramic in the range of 30 °C–90 °C is shown in Fig. 10 (b). It can be seen that the 0.90LLBNTZ-0.10NBN ceramic has high energy storage properties over a broad temperature range. The value of the energy storage density and energy storage efficiency is 0.91 J/cm^3^ and 79.51%, respectively, for 0.90LLBNTZ-0.10NBN ceramic at 100 kV/cm and 90 °C.

**Figure 11:**
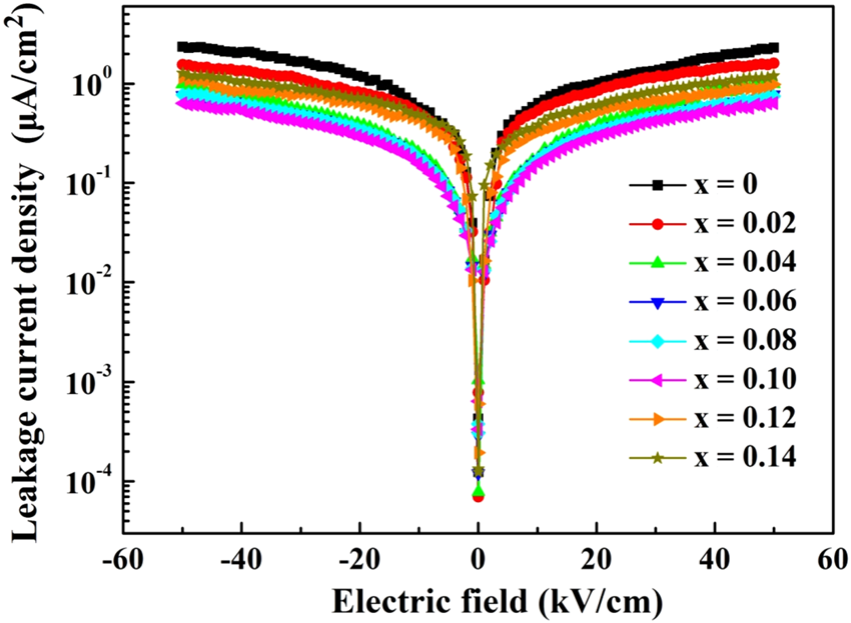
Leakage current density as a function of applied electric field for the (1-x)LLBNTZ-xNBN ceramics.

Insulating characteristic of the (1-x)LLBNTZ-xNBN ceramics were determined by using the value of leakage current density. Figure 11 presents the leakage current density as a function of applied electric field for the (1-x)LLBNTZ-xNBN ceramics with different x values measured at room temperature. The leakage current density of the samples increases gradually with increasing the applied electric field. The leakage current density of the samples possesses a radically increasing in the low electric field region, and a stabilized leakage current density is obtained with increasing the electric field. It can be found that the leakage current density of the (1-x)LLBNTZ-xNBN ceramics decrease with increasing the value of x from 0 to 0.10, which facilitates energy storage application. Whereas leakage current density is increased gradually with increasing the value of x when x ≥ 0.12. It is consistent with the result of BDS and energy storage properties for the (1-x)LLBNTZ-xNBN ceramics.

## Conclusions

A series of (1-x)LLBNTZ-xNBN ceramics were successfully fabricated via the conventional solid-state sintering methods. Their phase structure, microstructure, dielectric, ferroelectric and energy storage property were systematically investigated. The results indicate that double dielectric constant anomalies can be observed. The first dielectric constant anomaly peak shifts to lower temperature and the magnitude of the anomaly peak decreases with increasing the value of x. The second anomaly peak also decreases in magnitude while its position remains almost unchanged. All the samples have small values of leakage current density and the maximum energy storage density of 2.04 J/cm^3^ at 178 kV/cm can be obtained at room temperature. Energy storage properties over a broad temperature range can be obtained from 30 °C to 90 °C. The value of the energy storage density and energy storage efficiency is 0.91 J/cm^3^ and 79.51% respectively for the 0.90LLBNTZ-0.10NBN ceramic at 100 kV/cm and 90 °C. It can be concluded that the (1-x)LLBNTZ-xNBN ceramics are promising candidate materials for high temperature and energy storage over a broad temperature range applications.

## Methods

A series of (1-x)LLBNTZ-xNBN (x = 0–0.14) ceramics were prepared by the conventional solid-state sintering method. The first stage of the fabrication was the synthesis of Bi_0.48_La_0.02_Na_0.48_Li_0.02_Ti_0.98_Zr_0.02_O_3_ (LLBNTZ) and Na_0.73_Bi_0.09_NbO_3_ (NBN), respectively. Reagent grade Bi_2_O_3_ (>99%), La_2_O_3_ (>99.9%), Na_2_CO_3_ (>99.8%), Li_2_CO_3_ (>98%), TiO_2_ (>98%) and ZrO_2_ (>99%) were weighed according to the nominal composition of LLBNTZ and mixed for 12 h by ball milling in alcohol. After drying, the milled powders were calcined at 800 °C for 4 h in air, and then remilled in alcohol for 12 h. Reagent grade Bi_2_O_3_ (>99%), Na_2_CO_3_ (>99.8%), and Nb_2_O_5_ (>99.5%) were also weighed according to the nominal compositions of NBN and mixed for 12 h by ball milling in alcohol. After drying, the milled powders were calcined at 800 °C for 2 h in air, and then remilled in alcohol for 12 h. LLBNTZ and NBN powders were weighted according to the stoichiometric formula of (1-x)LLBNTZ-xNBN and mixed by ball milling in alcohol for 12 h. Afterwards, the suspensions were dried at 100 °C. Then obtained final powders were mixed with binders and uniaxially pressed into disk-shaped samples with 10 mm in diameter under a pressure of 200 MPa. And then the binders were burned out at 550 °C for 4 h, followed by sintering the samples at 1150 °C for 2 h. To prevent the loss of volatile Bi, Na, and Li, the green bodies were embedded in the corresponding powders during sintering.

X-ray diffractometer (XRD, D-MAX 2200 pc, Rigaku Co., Tokyo, Japan) was used to characterize the phase structure of the (1-x)LLBNTZ-xNBN ceramics. The microstructure of the polished and thermal-etched samples for the (1-x)LLBNTZ-xNBN ceramics was observed using SEM (S4800, Rigaku Co., Japan). For dielectric measurement, the sintered samples were polished to obtain smooth and parallel surfaces. Then, a silver paste was painted and fired at 800 °C for 20 min to form the electrode. The temperature dependent dielectric constant and dielectric loss were measured using the LCR meter (3532–50, Hioki, Ueda, Japan) in the frequency range from 1 kHz to 1 MHz. The *P-E* hysteresis loops and the leakage current density were characterized by a ferroelectric test system (Premier II, Radiant, USA) and the samples were immersed in silicone oil to avoid surface flashover in the process.

## Electronic supplementary material

Supplementary information
