# Supplementary material for: High energy storage density over a broad temperature range in sodium bismuth titanate-based lead-free ceramics
Source: Sci Rep. 2017 Aug 18;7:8726. doi: 10.1038/s41598-017-06966-7 (PMC5562768; doi:10.1038/s41598-017-06966-7)
Supplement: Supplementary file 1 — Supplementary information [file 41598_2017_6966_MOESM1_ESM.doc]

**On-line Supplementary information for**

High energy storage density over a broad temperature range in sodium bismuth titanate-based lead-free ceramics

Haibo Yang[[1]](#footnote-2)*, Fei Yan, Ying Lin, Tong Wang, Fen Wang

*School of Materials Science and Engineering, Shaanxi University of Science and Technology, 710021, Xi’an, PR China.

AUTHOR INFORMATION

Corresponding Author

(*Haibo Yang) E-mail: yanghaibo@sust.edu.cn.

Table S1 Temperature stability of the dielectric constant for (1-x)LLBNTZ-xNBN ceramics at 1 kHz.

| **Materials** | **Temperature range satisfying ∆C/C_150 °C_ ≤ ± 15%** | **Reference** |
| --- | --- | --- |
| 0.97(BNT-BT)-0.03NT | 77 ^o^C-356 ^o^C | 10 |
| 0.95(BNT-BT)-0.05NT | 66 ^o^C-336 ^o^C | 10 |
| 0.91(0.94BNT-0.06BT)-0.09KNN | 39 ^o^C-343 ^o^C | 44 |
| 0.94LLBNTZ-0.06NBN | 95 ^o^C-500 ^o^C | This work |
| 0.92LLBNTZ-0.08NBN | 76 ^o^C-500 ^o^C | This work |
| 0.90LLBNTZ-0.10NBN | 54 ^o^C-500 ^o^C | This work |
| 0.88LLBNTZ-0.12NBN | RT-500 ^o^C | This work |
| 0.86LLBNTZ-0.14NBN | RT-500 ^o^C | This work |

BNT: Bi_0.5_Na_0.5_TiO_3_, NT: NaTaO_3_, BT: BaTiO_3_, KNN: K_0.5_Na_0.5_NbO_3_, NBN: Na_0.73_Bi_0.09_NbO_3_, LLBNTZ: Bi_0.48_La_0.02_Na_0.48_Li_0.02_Ti_0.98_Zr_0.02_O_3_, RT: Room temperature.

Table S2 Energy storage properties of the (1-x)LLBNTZ-xNBN ceramics.

| **x** | **0** | | **0.02** | | **0.04** | | **0.06** | | **0.08** | **0.10** | **0.12** | **0.14** |
| --- | --- | --- | --- | --- | --- | --- | --- | --- | --- | --- | --- | --- |
| ***E* (kV/cm)** | 110 | 112 | | 112 | | 128 | | 157 | | 178 | 142 | 140 |
| ***W_1_* (J/cm^3^)** | 0.47 | 0.44 | | 0.92 | | 1.30 | | 1.86 | | 2.04 | 1.52 | 1.43 |
| ***η* (%)** | 17.27 | 17.61 | | 39.31 | | 49.75 | | 57.31 | | 54.76 | 62.55 | 69.18 |


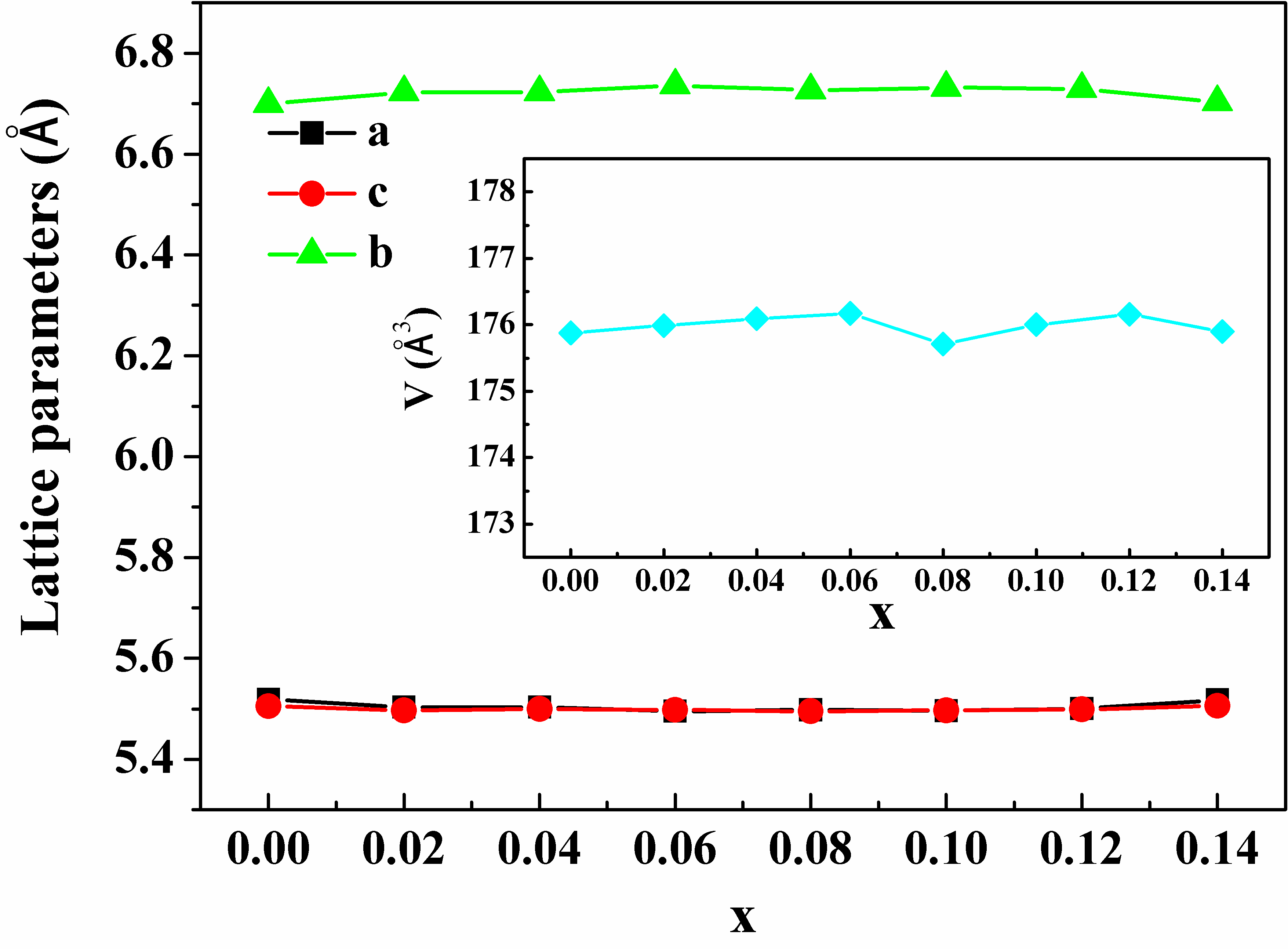


Fig. S1 Lattice parameters of the (1-x)LLBNTZ-xNBN ceramics and the inset figure shows the compositional dependence of the unit cell volume.


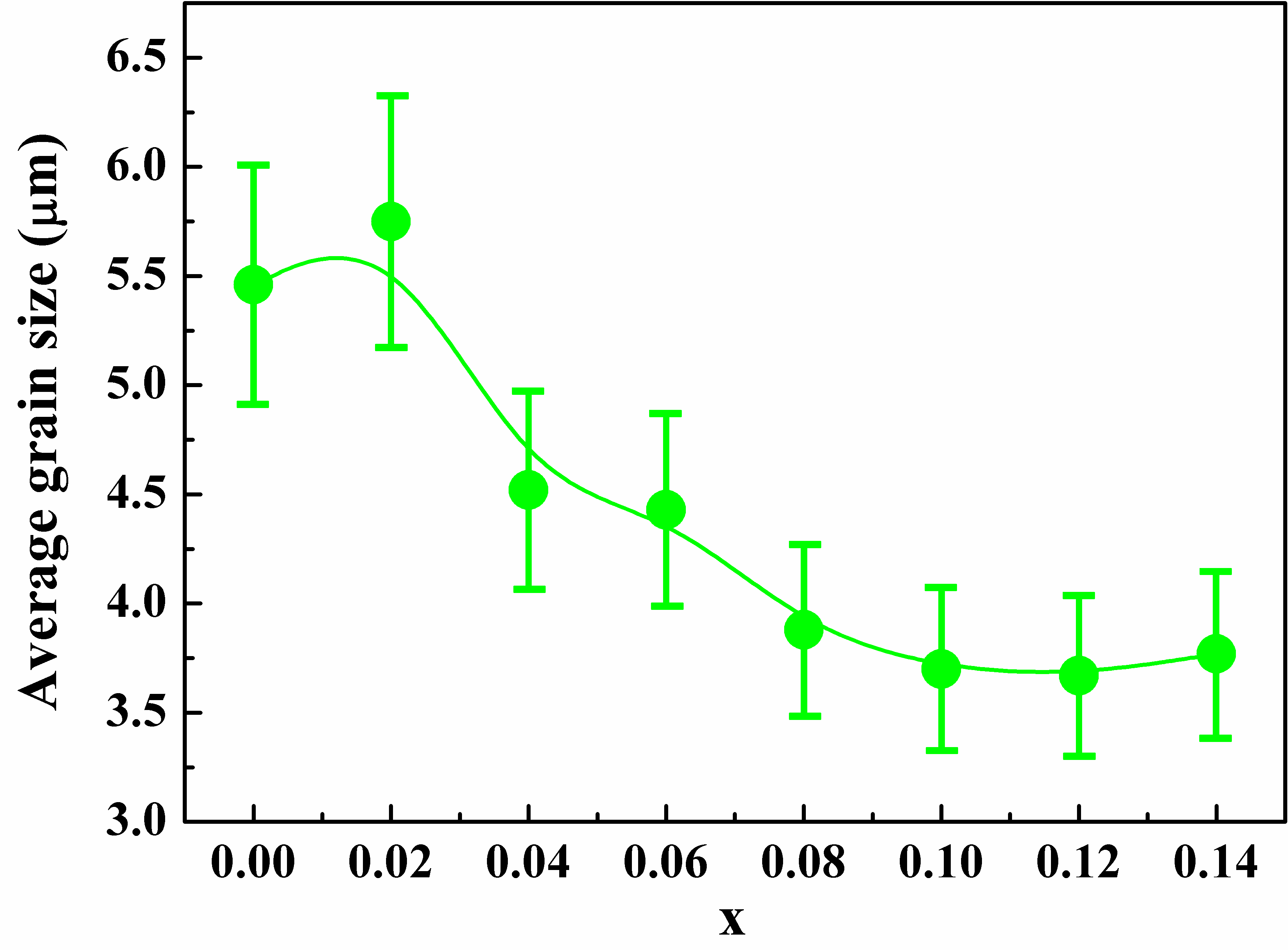


Fig. S2 The average grain size of (1-x)LLBNTZ-xNBN ceramics with different x value.


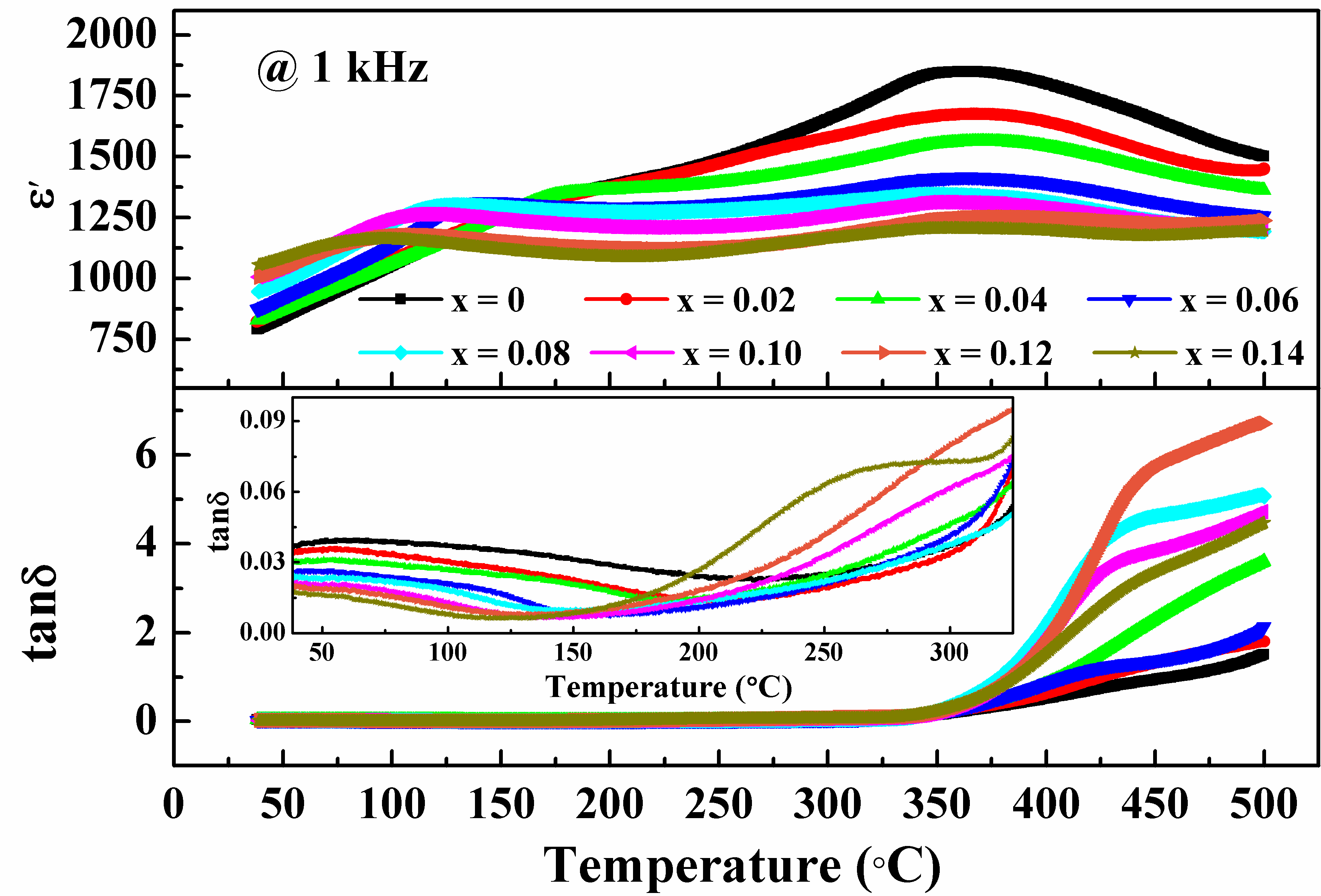


Fig. S3 Temperature dependence of dielectric constant and dielectric loss of the (1-x)LLBNTZ-xNBN ceramics measured at 1 kHz. (The inset shows dielectric loss of the (1-x)LLBNTZ-xNBN ceramics from room temperature to 325 ^o^C.)


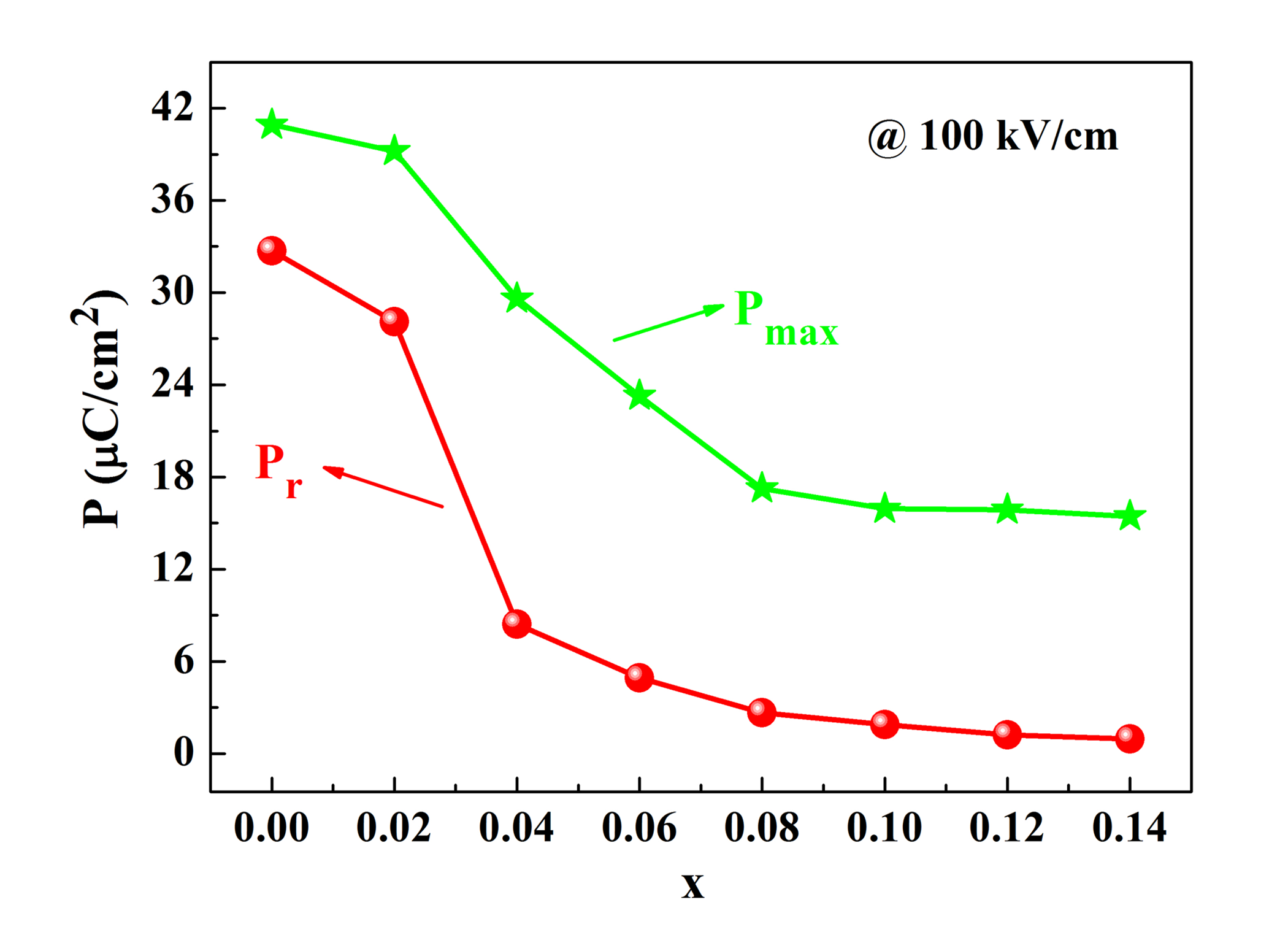


Fig. S4 The variation of *P_r_* and *P_max_* under 100 kV/cm estimated from *P-E* loops measured at room temperature and 10 Hz for the (1-x)LLBNTZ-xNBN ceramics.


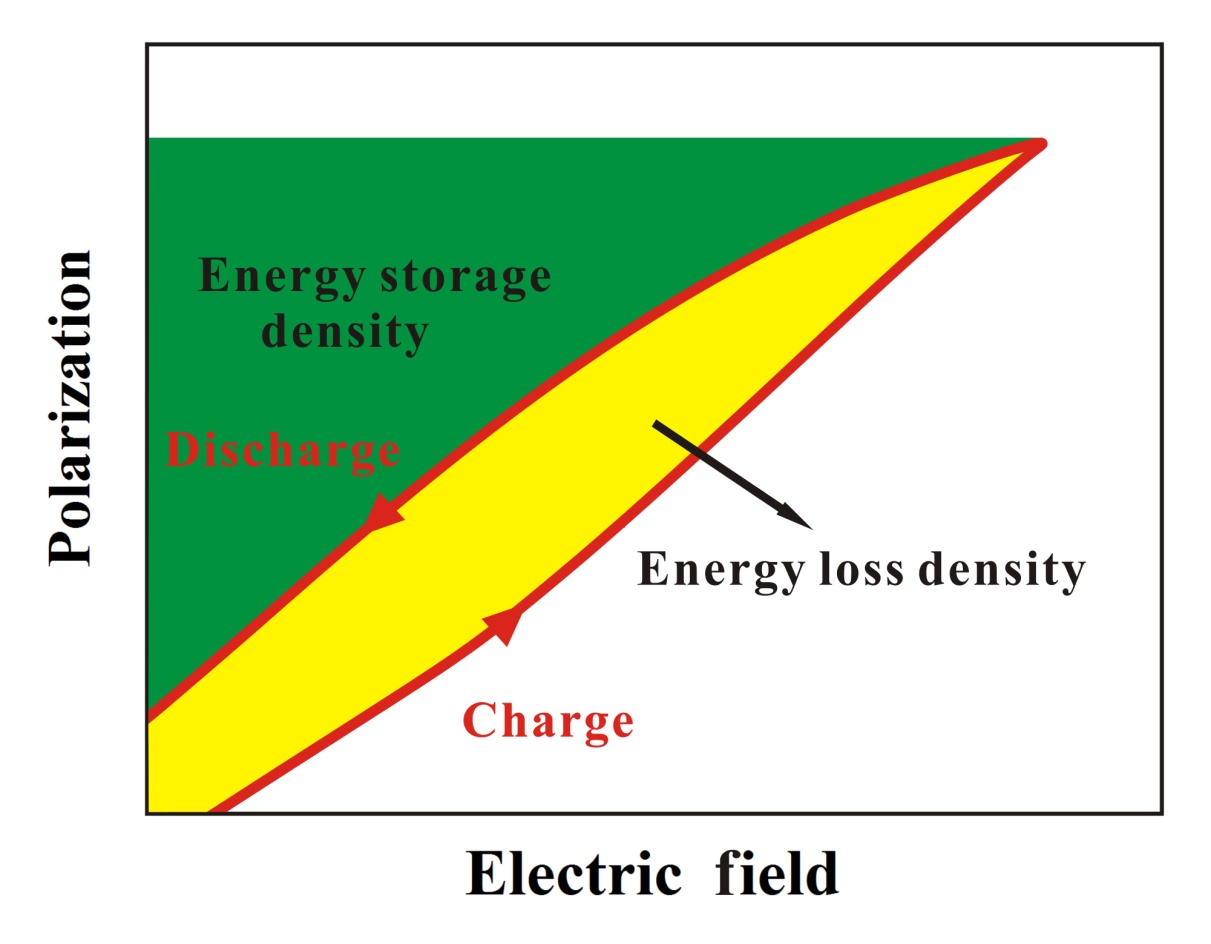


Fig. S5 Schematic diagram for the calculation of energy storage properties.

1. ^*^ Corresponding author. Tel: +86-29-86168688; Fax: +86-29-86168688; Email: yanghaibo@sust.edu.cn [↑](#footnote-ref-2)
